# Beyond Berlin: The multidimensional evolution of ARDS in the era of precision critical care

**DOI:** 10.1016/j.jointm.2026.02.004

**Published:** 2026-03-20

**Authors:** Ming Xue, Hui Chen, Ling Liu, Haibo Qiu

**Affiliations:** 1 Jiangsu Provincial Key Laboratory of Critical Care Medicine, Department of Critical Care Medicine, Zhongda Hospital, Southeast University, Nanjing, Jiangsu, China; 2 Department of Critical Care Medicine, The Fifth Affiliated Hospital of Xinjiang Medical University, Urumqi, Xinjiang, China

The paradigm of acute respiratory distress syndrome (ARDS) is undergoing a profound and multidimensional transformation. Moving beyond the Berlin definition, the recent global definition signifies not merely a diagnostic update but a strategic shift toward earlier recognition and intervention.^[^^1^^,^^2^^]^ Concurrently, advances in multi-omics are redefining ARDS as a heterogeneous disease with distinct molecular endotypes, while evidence generation is evolving from population-based averages toward individualized treatment prediction. This evolution, spanning diagnostic strategy, disease redefinition, evidence generation, ultimately effecting clinical practice (Figure 1), is driving ARDS toward a precision-care model that requires urgent and coordinated advancement.

**Figure 1:**
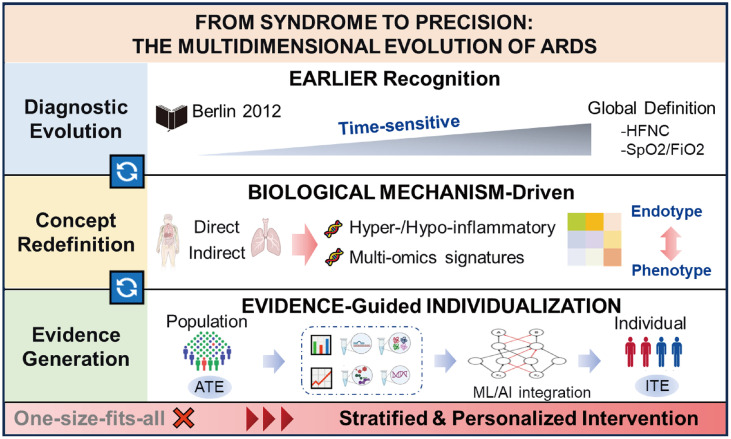
A conceptual framework for the multidimensional evolution of ARDS. AI: Artificial intelligence; ARDS: Acute respiratory distress syndrome; ATE: Average treatment effect; HFNC: High-flow nasal cannula; ITE: Individualized treatment effect; ML: Machine learning.

## Diagnostic Evolution

The new global ARDS definition embodies a paradigmatic reconceptualization of the syndrome—shifting our focus from rigid classification toward a dynamic, intervention-oriented framework. By deliberately expanding diagnostic inclusion to encompass patients receiving high-flow nasal oxygen and those in resource-limited settings, the definition prioritizes early sensitivity with a clear clinical intent: to reduce diagnostic delays and facilitate earlier initiation of lung-protective strategies aimed at preventing patient-self-inflicted lung injury (P-SILI). For non-intubated patients, such as those on high-flow nasal cannula, this includes vigilant monitoring of respiratory drive and mechanics, optimization of non-invasive support, consideration of adjunctive therapies like prone positioning, and timely reassessment for potential intubation when indicated.^[^^2^^]^ However, this necessary expansion also amplifies the inherent heterogeneity of the ARDS population, posing a persistent challenge: how to accurately distinguish ARDS from common confounders such as cardiogenic pulmonary edema, severe focal (e.g., lobar) pneumonia, or acute exacerbation of underlying interstitial lung disease in the absence of a definitive biomarker or gold-standard imaging modality. The LUNG SAFE study had previously highlighted this diagnostic tension, revealing that nearly half of mild ARDS cases were missed at the bedside—precisely the subgroup that stands to benefit most from timely protective management.^[^^3^^]^ Thus, the transition from the Berlin to the Global definition is not merely a change in criteria, but a strategic realignment of diagnostic philosophy: one that embraces earlier suspicion and intervention, while urgently calling for more precise phenotyping tools to guide therapy.

## Concept Redefinition

Beyond its clinical redefinition, ARDS is increasingly understood not as a uniform clinical syndrome, but as a multi-omics-driven disease characterized by distinct molecular subphenotypes.^[^^4^^]^ Traditional classifications based on direct versus indirect injury are now being supplemented—and often superseded—by endotypes such as the hyperinflammatory and hypoinflammatory profiles identified through transcriptomic, proteomic, and metabolomic analyses.^[^^5^^,^^6^^]^ These subphenotypes provide significant therapeutic implications: for instance, hyperinflammatory ARDS may respond favorably to higher PEEP strategies, conservative fluid management, or immunomodulation, whereas the same interventions may be ineffective or even harmful in hypoinflammatory patients.^[^^6^^,^^7^^]^ This evolution moves ARDS from a one-size-fits-all diagnostic model to a mechanism-based, treatable-trait framework, aligning critical care with the broader precision medicine paradigm.

The translation of these biological insights to the bedside hinges on the development of rapid, point-of-care phenotyping tools. Current efforts closest to clinical application include simplified prediction models using limited clinical variables and biomarkers (e.g., interleukin-6, bicarbonate),^[^^8^^]^ non-invasive respiratory effort monitoring technologies to assess the risk of patient-self-inflicted lung injury,^[^^9^^]^ and emerging point-of-care testing platforms for key proteins or metabolites.^[^^10^^,^^11^^]^ While comprehensive multi-omics profiling remains largely a research tool, these pragmatic approaches represent critical first steps toward real-time, actionable phenotyping.

## Evidence Generation

This profound biological heterogeneity erodes the cornerstone of evidence-based medicine—average treatment effects derived from randomized controlled trials (RCTs)—by exposing the gap between population means and individual biology. A systematic review of 67 ARDS RCTs revealed striking between-trial variability, with control-group 28-day mortality ranging from 9.7% to 66.7%—most of which remained statistically unexplained (I²=87.5%).^[^^12^^]^ Such variability underscores a critical limitation: the “average” trial patient is a statistical abstraction that often masks profound individual differences in treatment response. In response, the emerging paradigm of individualized treatment effect (ITE) estimation leverages machine learning and multi-dimensional data integration to predict which patients are most likely to benefit from specific interventions.^[^^13^^,^^14^^]^ This shift abandons population-level averages and embraces individualized medicine-explicitly accounting for inter-patient heterogeneity to deliver the right therapy to the right patient at the right time.

The practical implementation of this personalized approach is already yielding actionable insights, converting seemingly “negative” trials into springboards for individualized treatment guidance. In the BOUGIE trial, while the primary outcome was neutral, showing no average benefit for bougie versus stylet use in tracheal intubation, a causal forest algorithm identified a subgroup of patients who derived significant benefit from the bougie.^[^^15^^]^ Similarly, the PILOT trial found no overall mortality difference between higher and lower oxygen-saturation targets, but ITE analysis suggested that future strategies may require physiological or phenotypic stratification to optimize targets for individual patients. It is crucial to emphasize that these ITE findings are currently hypothesis-generating; they are designed to inform the design of future prospective, stratified trials rather than to mandate immediate deviation from standard care in clinical practice.^[^^14^^,^^16^^]^ Beyond ARDS, the EXIT-SEP trial in sepsis demonstrated that post-hoc phenotype-based analysis could identify specific subphenotypes (γ and δ) that gained substantial mortality benefit from Xuebijing injection—a finding invisible in the overall cohort analysis.^[^^17^^]^ These examples collectively show that data-driven, personalized decision-making is not a distant future but an emerging reality, capable of extracting clinically actionable insights even from ostensibly “negative” trials.

This paradigm shift—revising diagnostic rules, mapping biological diversity, and targeting therapy—requires a concerted translational drive to turn conceptual gains into quantifiable bedside benefit. Three parallel and synergistic initiatives are now imperative: first, the prospective, multi-center validation of the global definition across diverse healthcare settings to confirm its utility and refine its application; second, the development and implementation of rapid, point-of-care phenotyping tools capable of translating omics-derived subphenotypes into real-time, actionable clinical information; and third, a fundamental redesign of clinical trials towards phenotype-guided frameworks that prospectively validate interventions in predefined, mechanistically coherent patient subgroups. Ultimately, it is through this integrated triad—a sensitive and timely diagnostic definition, dynamic and accessible phenotyping, and intelligently targeted trial design—that we can navigate the profound heterogeneity of ARDS and realize the long-envisioned promise of precision critical care. The journey from syndrome to precision is now clearly charted; its realization demands a collective commitment from researchers, clinicians, and trialists alike.

## CRediT authorship contribution statement

**Ming Xue:** Writing – original draft, Conceptualization. **Hui Chen:** Writing – review & editing. **Ling Liu:** Writing – review & editing, Supervision, Conceptualization. **Haibo Qiu:** Writing – review & editing, Supervision, Conceptualization.
